# Vascular ageing manifestations and hypertension in the community

**DOI:** 10.1016/j.ajpc.2024.100918

**Published:** 2024-12-13

**Authors:** Guillermo A. Alanis, Pierre Boutouyrie, Mouad Abouqateb, Rosa Maria Bruno, Rachel E. Climie, Thomas van Sloten, Nicolas Danchin, Bruno Pannier, Stéphane Laurent, Xavier Jouven, Jean-Philippe Empana

**Affiliations:** a Université Paris Cité, INSERM U970, Paris Cardiovascular Research Centre, Team Integrative epidemiology of cardiovascular diseases, Paris, France; b Université Paris Cité, INSERM U970, Paris Cardiovascular Research Centre, Team Arterial diseases in women, Paris, France; c APHP, Georges Pompidou European Hospital, DMU CARTE, Department of Pharmacology, Paris, France; d Menzies Institute for Medical Research, University of Tasmanian, Hobart, Australia; e Department of Vascular Medicine, University Medical Center Utrecht, Utrecht, the Netherlands; f Investigations Précliniques de Paris (IPC), Paris, France

**Keywords:** Essential hypertension, Vascular ageing, Arteriosclerosis, Atherosclerosis, Echotracking

## Abstract

**Objective:**

To investigate the association between joint manifestations of vascular ageing (VA) and hypertension.

**Methods:**

We used baseline (2008–2012) and follow-up data (up to 2024) from the Paris Prospective Study III, a French cohort of 10,157 participants. Prevalent and incident hypertension were determined at baseline (blood pressure ≥140/90 mmHg or on medication) and at 2, 4, 6, 8 and 10 years of follow-up (self-reported antihypertensive treatment). VA manifestations were assessed at baseline via echo-tracking in the right common carotid artery. Clustering analysis identified patterns of VA and their association with hypertension was assessed with logistic regression.

**Results:**

The cross-sectional analysis included 9,096 participants (mean age: 59±6 years, 39 % female). Hypertension prevalence was 36 % (*n* = 3,276). Three clusters of VA manifestations were identified. Cluster 1 (*n* = 4,326;47.6 %) was characterized by healthy vascular ageing (HVA), Cluster 2 by increased arteriosclerosis (ART) (*n* = 2,274;25.0 %) and Cluster 3 by greater atherosclerosis prevalence (ATH) (*n* = 2,496;27.4 %). Compared to the HVA cluster, ART (aOR 3.94; 95 % CI 3.50;4.45) and ATH clusters (aOR 2.69; 95 % CI 2.38;3.04) were associated with prevalent hypertension. The prospective analysis included 5,310 normotensives with 754 (14.1 %) cases of incident hypertension (median follow-up of 10.05 years [range: 10.00;10.15]). Both ART (aOR 1.34; 95 % CI 1.08;1.65) and ATH (aOR 1.70; 95 % CI 1.40;2.07) clusters were associated with incident hypertension.

**Conclusion:**

Vascular ageing manifestations reflecting increased carotid arteriosclerosis and atherosclerosis are related to prevalent and incident hypertension.

## Graphical abstract

Association between clusters of vascular ageing manifestations with hypertension in the PPS3 study.

**Figure:**
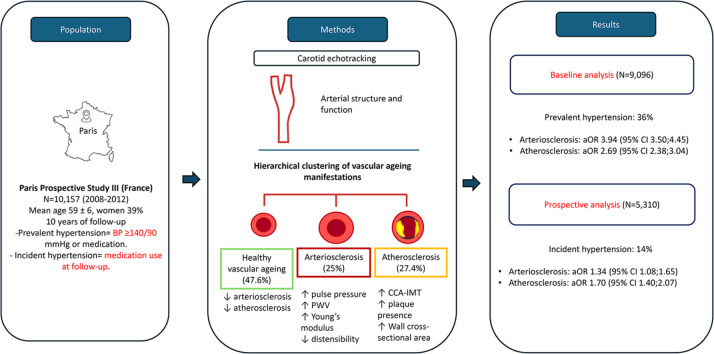

## 1 Introduction

High blood pressure (i.e., hypertension) is affecting 1.3 billion adults worldwide (33 % of adults aged 30–79) [1] with 7.8 million deaths per year attributed to high systolic blood pressure (>140 mmHg) [2]. Genetic, environmental, and traditional risk factors have been associated with hypertension [3]. Vascular ageing represents structural or functional changes in the vasculature that can reflect a cumulative lifetime exposure to these risk factors [4]. The relationship between vascular ageing, specifically arteriosclerosis and atherosclerosis, and hypertension is bidirectional: on one hand, elevated blood pressure not only induces vascular changes that increase arteriosclerosis [5,6] but is also associated with a greater incidence and progression of atherosclerosis [7]. On the other hand, changes in vascular ageing, reflecting either arteriosclerosis or atherosclerosis, precede and drive the development of hypertension. [[8], [9], [10], [11], [12]]

However, most studies investigating the relationship between vascular ageing and incident hypertension have focused on a single manifestation of vascular ageing related to arteriosclerosis (i.e., pulse wave velocity [PWV]) [[8], [9], [10]] or atherosclerosis (i.e., intima-media thickness [IMT]) [12]. Yet, the manifestations of vascular ageing are diverse and multidimensional [4]. How the different vascular aging manifestations cluster at the individual level, and whether individuals with different vascular aging patterns have different risk of hypertension remains unknown. Carotid echotracking is a high-resolution ultrasound technique that can simultaneously measure several vascular ageing manifestations related to both arteriosclerosis (local PWV, distensibility coefficient, young's modulus, β stiffness index, central pulse pressure, cross-sectional wall stress) and atherosclerosis (intima media thickness, plaque presence, wall-cross-sectional area, diameter). Therefore, the objective of this study was to investigate the joint association of several manifestation of vascular ageing reflecting arteriosclerosis and atherosclerosis with both prevalent and incident hypertension in the community. To this end, we used clustering analysis to identify patterns of vascular ageing manifestations among individuals. This question was addressed in the Paris Prospective Study III (PPS3).

## 2 Methods

### 2.1 Study population

The design, main objectives, and sample size calculation of the PPS3 have been previously described [13]. The baseline study population comprised 10,157 women and men from 50 to 75 years old who attended a preventive health facility (Centre d'Investigations Préventives et Cliniques) in Paris from 2008 to 2012. All participants underwent a complete clinical examination coupled with standard biological tests. A self-administered questionnaire gathered information about socioeconomic status, lifestyle, personal and family medical history, including CVD history (previous myocardial infarction, angina, or stroke), current health status, and use of medication. For the follow-up, study participants were evaluated every 2 years via self-administered questionnaire to assess change in risk factors and medications. At 10 years, the average retention rate was 83 % (ranged from 82 to 85 %). Each participant signed an informed consent. The study complied with the Declaration of Helsinki, was approved by the Ethics Committee of the Cochin Hospital (Paris) and is registered in the international trial registry (NCT00741728).

### 2.2 Vascular ageing assessment

Vascular manifestations related to arteriosclerosis, atherosclerosis and arterial remodeling were evaluated with echotracking (Artlab, Esaote, Maastritch, NL) following consensus guidelines [14]. 6-second clips were acquired on the right carotid artery at 10 mm proximal to the carotid bifurcation. Artlab software was used to measure systole diastolic changes in diameter and intima media thickness (IMT) as previously described [15]. Carotid pulse pressure (cPP), local carotid pulse wave velocity (cPWV), distensibility coefficient (DC), Young's elastic modulus, β stiffness index, wall-cross-sectional area (WCSA), and circumferential wall stress (CWS), were calculated. Formulas are included in Supplemental Methods. IMT was measured at the posterior wall of the common carotid artery in a plaque-free segment. Furthermore, right and left common and internal carotid arteries were scanned, and presence of plaque was reported as a discrete variable.

### 2.3 Prevalent and incident hypertension

Blood pressure was assessed twice at the same visit. In the first assessment which takes place at the start of the care pathway at the IPC centre, blood pressure was measured three times in a sitting position after 10 min of rest in both arms using a validated oscillometric device (Omron 705 C, Kyoto, Japan) with an appropriately sized cuff. In the second assessment which takes place at the end of the care pathway at the IPC centre, blood pressure was measured once in the right arm, after a 10-minute supine rest, before carotid echotracking evaluation. This second blood pressure measurement was considered as baseline blood pressure to reflect a true resting hemodynamic state and also because it was used for vascular ageing parameter calculation. . Baseline blood pressure levels were categorized according to the 2023 European Society of Hypertension guidelines as follows: optimal (<120 and <80 mmHg), normal (120–129 and 80–84 mmHg), high normal (130–139 mmHg and/or 85–89 mm), and ≥G1 hypertension (≥140 and/or ≥90 mmHg) [16]. Prevalent hypertension was defined as a blood pressure ≥140/90 mmHg or current use of antihypertensive medication. The class of antihypertensive medications were identified using the Anatomical Therapeutic Chemical (ATC) codes including diuretics (C03) (including thiazides(-like), loop and potassium sparing diuretics), beta-blockers (C07), calcium channel blockers (C08), agents acting on the renin-angiotensin system (C09). Medications coded as C02 (*n* = 52) (antiadrenergic agents with central, peripheral of ganglion-blockers activity) were not included due to their direct vasodilating effect that could alter the measurement some vascular ageing manifestations (e.g., arterial diameter, distensibility coefficient). Hypertension during follow-up was defined as self-reported antihypertensive medication use at follow-up.

### 2.4 Baseline covariates

Smoking status distinguished between current and non-current smokers. Alcohol intake was categorized as abstain, occasional (certain days of the week) and regular consumption (daily or almost daily). Education was binarized as basic (high school or lower) or high education (bachelor or higher). Body mass index (BMI) was calculated as kg/m2 based on measured height and weight. Diabetes was defined as a fasting glucose concentration of ≥126.0 mg/dl (7.0 mmol/l), a non-fasting glucose concentration of ≥200.0 mg/dl (11.1 mmol/l), or the use of anti-diabetic treatment. The glomerular filtration rate was estimated (eGFR) using the CKD-EPI equation [17].

### 2.5 Statistical analysis

#### 2.5.1 Clustering of vascular ageing manifestations

Hierarchical clustering using Wald distance was employed to identify patterns of vascular ageing aiming to maximize the intra-clusters similarities while minimizing inter-clusters similarities. Vascular ageing parameters were processed by principal component analysis to correct for multicollinearity retaining six dimensions that explained 98.37 % of the total inertia. These dimensions were used for hierarchical agglomerative clustering with Euclidean distance measures and Ward's linkage criterion [18]. The optimal number of clusters was chosen based on clinical and statistical criteria. Clinical criteria consider the number of participants by cluster together with a number of clusters that is clinically usable. Regarding the statistical criteria, we allowed the number of clusters to vary the number of clusters between 2 and 6 and calculated 26 clustering validity indices using the NbClust R package version 3.0.1. The selected number of clusters corresponded to the one with the highest rates of validity indices (see online Supplementary methods).

#### 2.5.2 Main analysis

In descriptive analysis, numerical variables are presented as mean (SD), unless stated otherwise and categorical variables as proportions.

#### 2.5.3 Cross-sectional analysis

The association of clusters of vascular ageing (main exposure) with prevalent hypertension (outcome) was investigated using binary logistic regression, while their association with blood pressure categories and with the number of antihypertensive medications (0, 1, 2 and more) was evaluated using multinomial and ordinal logistic regression, respectively. Linear regression analysis was conducted when associating clusters of vascular ageing with considering systolic and diastolic blood pressure level.

#### 2.5.4 Prospective analysis

This analysis was conducted among those who were free of hypertension at baseline. Follow-up was censored at the first follow-up when a participant reported taking antihypertensive medication (for event) or at the last questionnaire or at the time of death which ever came first (for the non- event). Binary and ordinal logistic regression analyses were used to assess the association of clusters of vascular ageing with hypertension and the number of antihypertensive medications use at follow-up, respectively.

In both the prevalent and incident analyses, multivariate regression models were adjusted for age, sex, education, BMI, smoking, alcohol intake, diabetes, lipid-lowering drugs, high-density and total cholesterol, eGFR, and heart rate at baseline, and used the healthy vascular ageing cluster (cluster 1) as the reference category. To evaluate whether associations differed by age and sex, interaction term between age, sex and the clusters of vascular age was included separately in the models.

#### 2.5.5 Secondary and sensitivity analysis

In secondary analysis, the association between individual manifestation of vascular ageing and prevalent and incident hypertension was investigated in separate multivariable regression analyses. Several sensitivity analyses were performed. First, to control for indication bias related to antihypertensive medication prescription at follow-up, propensity score analysis was conducted (See Supplemental Methods for details). Second, given that participants with previous CVD could already present with accelerated vascular ageing, the analysis of prevalent and incident hypertension was repeated after excluding those with a history of CVD at baseline. Third, because antihypertensive medications can be prescribed for diagnoses other than hypertension (e.g., heart failure, post myocardial infarction), the prospective analysis was repeated after excluding those who experienced a cardiovascular disease event during follow-up. Fourth, to address residual confounding due to traditional risk factors, the analyses were repeated in the subsample of participants with no major comorbidities. Fifth, due to the relationship between peripheral pulse pressure and hypertension, the association between vascular ageing clusters and prevalent and incident hypertension was further adjusted for brachial pulse pressure. Lastly, the association between clusters of vascular ageing manifestations and incident hypertension was stratified by baseline blood pressure categories. All analyses and figures were conducted using R (2023.06.2). A two-tailed p-value <0.05 was considered statistically significant.

## 3 Results

### 3.1 Study sample

**Fig. 1:**
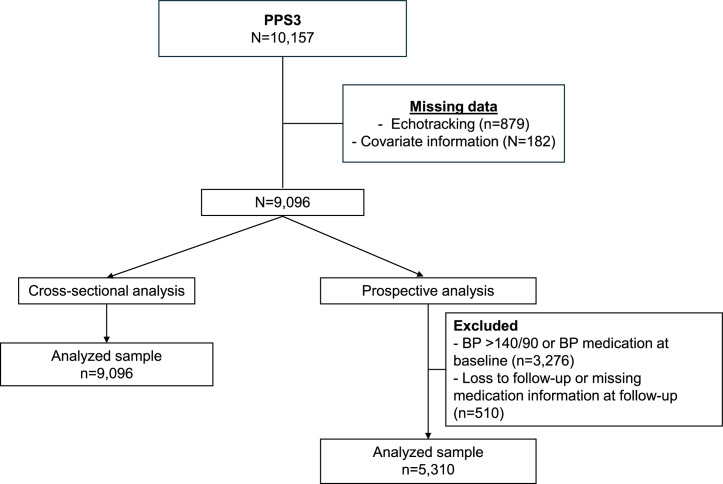
Study flow chart.

The study flow chart is shown in Fig. 1. From the original sample (*n* = 10,157), after excluding those with missing carotid echotracking assessments (*n* = 879) and covariates (*n* = 182) the study sample comprised 9,096 study participants. Demographics, blood pressure and other traditional risk factors did not differ between excluded and the analyzed population. (Supplemental Table 1).

### 3.2 Cluster of vascular ageing

We identified three clusters of manifestations of vascular ageing (Supplemental Methods; Supplemental Table 13). Cluster 1 (*n* = 4326, 47.6 %) was characterized by the lowest indicators of arteriosclerosis (lowest cPWV, β stiffness index, cPP, Young's modulus, CWS, and highest DC), atherosclerosis and structural remodeling (lowest CCA-IMT, WCSA, diameter, and prevalence of carotid plaques) and was labelled as the healthy vascular ageing (HVA) cluster. Cluster 2 (*n* = 2274, 25 %) had the highest arteriosclerosis parameters (highest cPWV, β stiffness index, cPP, Young's modulus, CWS, and low DC), and relatively low indicators of atherosclerosis, and was labelled as the arteriosclerosis (ART) cluster. Cluster 3 (*n* = 2496, 27.4 %) had the highest indicators of atherosclerosis (highest IMT and prevalence of plaques) and greater structural remodeling (higher diameter, WCSA) with lower levels of arteriosclerosis compared to Cluster 2 and was labelled as the atherosclerosis (ATH) cluster. The individual distribution of vascular ageing manifestations and clinical characteristics by cluster are presented in **Supplemental Table S2-S3.**

### 3.3 Study sample characteristics

The baseline characteristics of the study population by blood pressure category are shown in Table 1. The mean age (SD) was 60 (6) years and 39 % were females. As baseline blood pressure levels increased so too did age, BMI, and the prevalence of alcohol consumption, current smoking, CVD, type 2 diabetes, and antihypertensive medication use. In the total population, ACEI/ARB was the most frequent antihypertensive medication used (8.7 %) while diuretics (1.4 %) were the least frequently used. Most participants took one antihypertensive medication (11 %). In addition, the prevalence of the clusters reflecting arteriosclerosis and atherosclerosis increased with blood pressure categories.

**Table 1:** Characteristics of study participants by blood pressure categories at baseline.

	Optimal, <120 and <80 mmHg *N* = 2099	Normal, 120–129 and 80–84 mmHg *N* = 2401	High Normal, 130–139 and/or 85–89 mmHg *N* = 2084	≥Grade 1 hypertension, ≥140 and/or ≥90 mmHg *N* = 2512	Overall, *N* = 9096
Age, years	58 (6)	59 (6)	60 (6)	61 (7)	60 (6)
Sex, male	969 (46 %)	1540 (64 %)	1394 (67 %)	1653 (66 %)	5556 (61 %)
BMI, kg/m^2^	23.8 (3.4)	24.9 (3.4)	25.6 (3.6)	26.1 (3.7)	25.1 (3.6)
HDL, mg/dL	60 (16)	58 (15)	57 (15)	58 (15)	58 (15)
Total cholesterol, mg/dL	219 (36)	221 (36)	220 (35)	223 (37)	221 (36)
Systolic BP, mmHg	112 (6)	124 (3)	134 (3)	151 (12)	131 (16)
Diastolic BP, mmHg	67 (6)	73 (6)	77 (6)	85 (10)	76 (10)
eGFR, mL/min/1.73 m^2^	85 (12)	85 (12)	83 (12)	82 (13)	84 (12)
History of CVD	46 (2.2 %)	40 (1.7 %)	40 (1.9 %)	69 (2.7 %)	195 (2.1 %)
Type 2 diabetes	46 (2.2 %)	73 (3.0 %)	96 (4.6 %)	154 (6.1 %)	369 (4.1 %)
Alcohol intake					
Abstainer	287 (14 %)	277 (12 %)	242 (12 %)	302 (12 %)	1108 (12 %)
Occasional	1418 (68 %)	1563 (65 %)	1299 (62 %)	1458 (58 %)	5738 (63 %)
Regular	394 (19 %)	561 (23 %)	543 (26 %)	752 (30 %)	2250 (25 %)
Smoking					
Non-smoker	1134 (54 %)	1265 (53 %)	1082 (52 %)	1246 (50 %)	4727 (52 %)
Former	573 (27 %)	775 (32 %)	714 (34 %)	874 (35 %)	2936 (32 %)
Active	392 (19 %)	361 (15 %)	288 (14 %)	392 (16 %)	1433 (16 %)
Antihypertensive medications					
Beta-blockers	59 (2.8 %)	91 (3.8 %)	92 (4.4 %)	153 (6.1 %)	395 (4.3 %)
CCB	19 (0.9 %)	34 (1.4 %)	62 (3.0 %)	139 (5.5 %)	254 (2.8 %)
ACEI/ARB	78 (3.7 %)	141 (5.9 %)	213 (10 %)	362 (14 %)	794 (8.7 %)
Diuretics	22 (1.0 %)	27 (1.1 %)	32 (1.5 %)	43 (1.7 %)	124 (1.4 %)
Number of antihypertensive medications use					
0	1956 (93 %)	1957 (78 %)	1765 (85 %)	2162 (90 %)	7840 (86 %)
1	112 (5.3 %)	433 (17 %)	248 (12 %)	191 (8.0 %)	984 (11 %)
≥2	31 (1.5 %)	122 (4.9 %)	71 (3.4 %)	48 (2.0 %)	272 (3.0 %)
Clusters of vascular ageing					
HVA	1531 (73 %)	1416 (59 %)	845 (41 %)	534 (21 %)	4326 (48 %)
ART	210 (10 %)	428 (18 %)	575 (28 %)	1061 (42 %)	2274 (25 %)
ATH	358 (17 %)	557 (23 %)	664 (32 %)	917 (37 %)	2496 (27 %)

Data are mean (SD) or n(%). Blood pressure categories followed the 2023 ESH guidelines [16]. ACEI, angiotensin converting enzyme inhibitor; ARB, angiotensin receptor blocker; ART, arteriosclerosis; ATH, atherosclerosis; BMI, body mass index; BP, blood pressure; CCB, calcium channel blockers; CVD, cardiovascular disease; eGFR, estimated glomerular filtration rate; HDL, high-density cholesterol; HVA, healthy vascular ageing.

### 3.4 Vascular ageing and prevalent hypertension

**Fig. 2:**
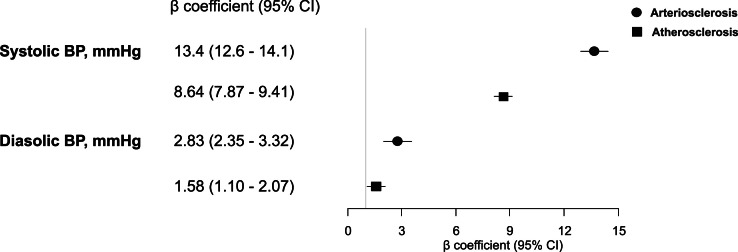
Association between clusters of vascular ageing manifestations and blood pressure (BP) levels at baseline. Coefficients were estimated with multivariate linear regression and using the healthy vascular ageing cluster as the reference category. Coefficients were adjusted for age, body mass index, diabetes, smoking, education, alcohol intake, total cholesterol, high-density cholesterol, lipid lowering drugs, estimated glomerular filtration rate, and heart rate. Association with systolic BP and with diastolic BP was systematically higher for the arteriosclerosis cluster as compared the atherosclerosis cluster (p for comparison<0.001).

The associations of the clusters of vascular aging manifestations with hypertension status and blood pressure categories at baseline are reported in Table 2, whereas associations with SBP and DBP at baseline are plotted in Fig. 2 and supplemental Table S4. The odds of prevalent hypertension were four-fold and more than two-fold higher in ART and ATH compared to HVA, respectively. ART and ATH compared to HVA had a graded association with the categories of blood pressure levels (p for trend <0.001), with stronger associations with ART as compared to ATH. Participants from ART and ATH, compared to HVA, had on average much higher SBP (β=13.4 mmHg; 95 % CI 12.6–14.1 and β=8.64 mmHg; 95 %CI 7.87–9.41, respectively) and DBP (β=2.83 mmHg; 95 % CI 2.35–3.32 and β=1.58 mmHg; 95 %CI 1.10–2.07, respectively). The association between the clusters of vascular ageing manifestations and prevalent hypertension did not differ by sex (p for interaction=0.329) nor by age (p for interaction=0.087).

**Table 2:** Association between clusters of vascular ageing manifestations and hypertension status and blood pressure categories at baseline.

		Blood pressure categories†				
	Prevalent hypertension* *n* = 3276	Optimal (<120 and <80 mmHg) (*n* = 2099)	Normal (120–129 and 80–84 mmHg) (*n* = 2401)	High normal (130–139 mmHg and/or 85–89 mm) (*n* = 2084)	≥Grade 1 (≥140 and/or ≥90 mmHg) (*N* = 2512)	
	OR (95 % CI)					
HVA, *n* = 4326	Ref.	Ref.	Ref.	Ref.	Ref.	P for trend <0.001
ATH, *n* = 2496	2.69 (2.38;3.04)	–	1.36 (1.15;1.60)	2.48 (2.10;2.94)	4.79 (4.04;5.69)	
ART, *n* = 2274	3.94 (3.50;4.45)	–	1.97 (1.63;2.38)	4.04 (3.35;4.88)	10.41 (8.62;12.56)	
	*P* < 0.001 ‡	p for difference between ART and ATH clusters≤0.01				

Blood pressure categories followed the 2023 ESH guidelines [16]. *Multivariate binary logistic regression. ^†^Multinomial logistic regression with HVA and optimal BP as the reference categories. ‡Indicates that the association with hypertension is stronger with ART as compared to ATH. P for trend derived from ordinal logistic regression and suggests that ART and ATH had a graded association with BP categories. Results were adjusted for age, sex, body mass index, diabetes, smoking, education, alcohol intake, lipid-lowering drugs, high-density and total cholesterol, estimated glomerular filtration rate, and heart rate. ART, arteriosclerosis; ATH, atherosclerosis; BP, blood pressure; HVA, healthy vascular ageing.

### 3.5 Vascular ageing and incident hypertension

**Fig. 3:**
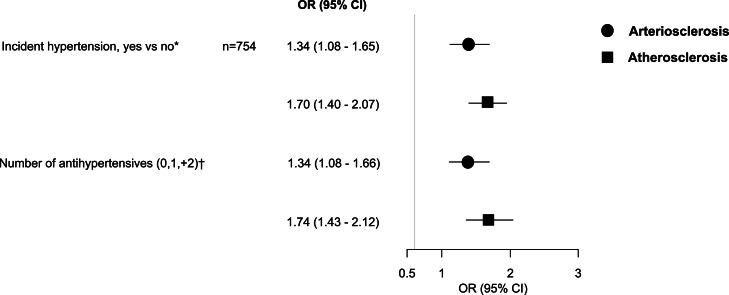
Association between clusters of vascular ageing manifestations and hypertension and number of medications at follow-up. ^a^ multivariate logistic regression. ^b^ ordinal logistic regression. Healthy vascular ageing was the reference category in all regression analyses. Results adjusted for age, body mass index, diabetes, smoking, education, alcohol intake, total cholesterol, high-density cholesterol, lipid-lowering drugs, estimated glomerular filtration rate, and heart rate. ACE, angiotensin converting enzyme; ARB, angiotensin receptor blocker. The association with hypertension and number of medications at follow-up did not differ between arteriosclerosis (circle) and atherosclerosis(square) (*p* = 0.104 and *p* = 0.082, respectively).

After excluding participants with baseline hypertension (*n* = 3276) and with missing follow-up medication information (*n* = 510), the study population included 5310 participants. Excluded participants only differed from the analyzed population by presenting a greater prevalence of previous CVD (Supplemental Table 1). After a median follow-up of 10.05 years (10.00 - 10.15 years), 754 (14.2 %) participants reported taking antihypertensive medications. Among them, the most frequently used was ACE/ARB medication (57 %) while the least frequently used medication was diuretics (8.6 %). Most participants used one (72 %) antihypertensive medication at follow-up. The distribution of classes and number of medications during follow-up are shown in **Supplemental Figure 2–3.** The multivariable associations between the clusters of vascular ageing manifestations and incident hypertension are plotted on Fig. 3. Compared with HVA, ART and ATH were associated with incident hypertension (adjusted OR 1.34, [95 % CI 1.08–1.65] and 1.70 [95 % CI 1.40;2.07], respectively). Also, ART and ATH compared to HVA were similarly associated with the number of antihypertensive medications at follow-up. The association of ART and ATH with incident hypertension decreased with age (p for interaction with age=0.027; Supplemental Table S5). Also, the association differed by sex, so that association with ART was seen only in women, whereas association with ATH was seen in both men and women (p for sex interaction=0.023, Supplemental Table S6).

### 3.6 Secondary and sensitivity analysis

When analyzed individually, each manifestation of vascular ageing was associated with hypertension and blood pressure at baseline on the one hand, and with incident hypertension on the other hand. (Supplemental Table S7) In sensitivity analysis, association with incident hypertension remained consistent after propensity score analysis that accounts for indication bias related to antihypertensive medication prescription (Supplemental Table S8-S9). Similarly, association with prevalent and incident hypertension remained consistent after excluding participants who had prevalent CVD or those who experienced a CVD event during follow-up, and among participants with no major cardiovascular risk factors at baseline (Supplemental Table S10-S11). Stratified analyses show in particular that the association of ATH with incident hypertension was seen even among participants with optimal blood pressure (Supplemental Table S11). Furthermore, after adjusting for peripheral pulse pressure, the association of ART and ATH with prevalent and incident hypertension attenuated but remained consistent with the main analysis (Supplemental Table S12).

## 4 Discussion

In this community-based study of 9,096 study participants who underwent high-precision carotid echo-tracking, we identified three clusters of vascular ageing manifestations characterized by healthy vascular ageing, arteriosclerosis, or atherosclerosis. Compared to healthy vascular ageing, arteriosclerosis and atherosclerosis clusters were associated both with prevalent hypertension and incident hypertension over 10-years of follow-up.

Several community-based studies have investigated the relationship between vascular ageing manifestations and prevalent and incident hypertension [8,9,12,19]. These studies have mostly focused on a single parameter and dimension of vascular ageing. In the Framingham Offspring cohort study, baseline aortic stiffness as measured by carotid-femoral PWV was associated with blood pressure progression and incident hypertension (OR 1.3 [1.0–1.6]) over a 7-year follow-up period [9]. Focusing on atherosclerosis, the Elsa-Brazil [12] and KoGes studies, [11] found an association between carotid IMT and incident hypertension. The ARIC study showed individual associations between different parameters of carotid stiffness (β stiffness index, diameter change, Young's, and Peterson's elastic modulus) and incident hypertension [19]. In the MESA study, lower aortic distensibility and higher carotid IMT levels were incrementally associated with incident hypertension after a 4.3-year follow-up period [20]. Our study replicates the association of individual vascular ageing manifestations with hypertension made in these previous studies. Furthermore, this is the first large study identifying patterns of vascular ageing based on 10 simultaneously measured vascular ageing parameters. The distribution of the individual manifestation of vascular ageing and of the CVD risk factors across these clusters demonstrate the relevance of these clusters. Some degree of overlapping between ART and ATH is expected given the well-known interrelation between arteriosclerosis and arteriosclerosis [21]. The association between the clusters of vascular ageing manifestations and hypertension was seen in multivariate analysis and confirmed in a number of sensitivity analyses addressing in particular residual confounding and indication bias, supporting the robustness of our findings.

Several mechanisms have been described to explain the relationship between vascular ageing and increased blood pressure. Arteriosclerosis can increase blood pressure by a) reduced pressure cushioning during systole and b) earlier arrival of the backward pulse wave increasing systolic pressure [6]. Atherosclerosis could reflect an inflammatory environment associated with endothelial dysfunction, decreased dilation capacity, and increased vascular resistance, that among other factors, contribute to the development of hypertension [22]. Regarding the sex differences in the association between vascular ageing and incident hypertension observed in our study, differences in vascular phenotypes has been previously reported between men and women [23]. The association between the arteriosclerosis cluster and incident hypertension only in women in our study, could be explained by a greater arterial stiffening with age in women compared to men [24].

The study contributes to suggest that vascular ageing assessment could help identifying individuals with a higher risk of developing hypertension beyond traditional risk factors measurements. In this context, the increased odds of incident hypertension associated with the clusters of arteriosclerosis and atherosclerosis among those with optimal blood pressure is of particular interest. The detection of accelerated vascular ageing may call for more intensive non-pharmacological (e.g., weight loss, physical activity) or pharmacological intervention strategies aimed at improving arterial health. For example, meta-analysis of clinical trials showed that aerobic exercise and high-intensity interval training can improve arteriosclerosis [25]. Regarding pharmacological interventions, the SPARTE trial showed that targeting arteriosclerosis in addition to blood pressure per se reduced SBP more compared to only targeting blood pressure (−1.08 mmHg/year vs −0.10 mmHg/year; *p* = 0.001, respectively) [26]. Other clinical trials have reported that the use of statins (rosuvastatin), proprotein convertase subtilisin/kexin type 9 inhibitors (PCSK9), and some anti-inflammatory drugs (colchicine) have the potential to stop the progression or even regress atherosclerosis. [[27], [28], [29]]

Due to lack of ambulatory/home blood pressure measurements, we were not able to distinguish participants with white-coat (WCH) or masked hypertension. WCH is a condition where there is an elevated office (≥140/90 mmHg) and normal out-of-office blood pressure (home/ambulatory daytime <135/85 mmHg). Conversely, masked hypertension is defined as normal office (<140/90 mmHg) and elevated out-of-office blood pressure (home/ambulatory daytime ≥135/85 mmHg) [30]. WHC and masked hypertension have a prevalence of 15 % and 10 % in the general population, respectively, and both conditions are associated with progression to sustained hypertension [31] In this study, WCH might have caused an overestimation of hypertension prevalence in the cross-sectional analysis. In the prospective analysis, participants with WHC could have been excluded due to elevated office blood pressure (>140/90 mmHg), while the effect of masked hypertension on incident hypertension could not be accounted for. In the prospective analysis, incident hypertension was defined as self-reported use of antihypertensive medication, which may not have captured a significant proportion of cases, as only 48.9 % of hypertensives in France are treated [32]. Self-reported hypertension is highly specific (89.5 %; 95% CI 84.0–93.3 %) but has low sensitivity (42.1 %; 95 % CI 30.9–54.2 %) [33]. Despite this, due to its low cost and ease of use it is a valuable tool employed in several large epidemiological studies. For example, the CDC uses the Behavioral Risk Factor Surveillance System, based on self-reported hypertension and antihypertensive medication intake, to estimate changes in hypertension prevalence [34]. In the ARIC study, self-reported use of blood pressure medication was considered to study the association between glycated hemoglobin and incident hypertension [35]. Excluded participants in this study comprised 283 lost-to-follow-up and 227 with missing follow-up medication data with similar demographic and risk factor characteristics compared to the analyzed population. Even though selection bias is unlikely, this could have had an impact on statistical power. Other limitations of our study include missing serial carotid echotracking assessments to examine the association between changes in vascular ageing and incident hypertension. Lastly, this study was based on a French and essentially Caucasian population with an age range of 50–75 years. Thus, our findings may not be generalizable to other ethnic and age groups.

## 5 Conclusions

To conclude, we have identified three clusters of vascular ageing based on structural/functional characteristics as: healthy vascular ageing, arteriosclerosis, and atherosclerosis. Compared to the healthy vascular ageing cluster, arteriosclerosis and atherosclerosis clusters were associated with prevalent and incident hypertension status. Further studies are needed to validate these findings in different populations.

## Data availability

The data that support the findings of the Paris Prospective Study III study cannot be shared publicly due to the privacy of individuals who participated in the study. However, pseudo-anonymized data can be made available from the corresponding author upon reasonable request, pending evaluation by the Paris Prospective Study III scientific committee of the research application.

### Sources of funding

The PPS3 Study was supported by grants from the National Research Agency (ANR), the Research Foundation for Hypertension (FR-HTA), the Research Institute in Public Health (IRESP), the Region Ile de France (Domaine d'Intérêt Majeur), the INSERM International Research Project grant, and a European Horizon H2020 grant. Dr. Alanis was funded by the scholarship “Talento Global” provided by the Universidad de Guadalajara, Mexico.

## CRediT authorship contribution statement

**Guillermo A. Alanis:** Formal analysis, Visualization, Writing – original draft. **Pierre Boutouyrie:** Writing – review & editing, Conceptualization. **Mouad Abouqateb:** Writing – review & editing, Methodology, Data curation. **Rosa Maria Bruno:** Writing – review & editing, Methodology. **Rachel E. Climie:** Writing – review & editing. **Thomas van Sloten:** Writing – review & editing. **Nicolas Danchin:** Writing – review & editing, Conceptualization. **Bruno Pannier:** Writing – review & editing, Investigation. **Stéphane Laurent:** Writing – review & editing, Conceptualization. **Xavier Jouven:** Writing – review & editing, Supervision, Funding acquisition, Conceptualization. **Jean-Philippe Empana:** Writing – review & editing, Validation, Supervision, Funding acquisition, Conceptualization.

## Declaration of competing interest

The authors declare the following financial interests/personal relationships which may be considered as potential competing interests:

Jean-Philippe Empana reports financial support was provided by National Research Agency (ANR). Jean-Philippe Empana reports financial support was provided by European Horizon 2020. Jean-Philippe Empana reports financial support was provided by Research Foundation for Hypertension (FR-HTA). If there are other authors, they declare that they have no known competing financial interests or personal relationships that could have appeared to influence the work reported in this paper.

## Appendix Supplementary materials

Image, application 1
