# Supplementary material for: Vascular ageing manifestations and hypertension in the community
Source: Am J Prev Cardiol. 2024 Dec 13;21:100918. doi: 10.1016/j.ajpc.2024.100918 (PMC11728902; doi:10.1016/j.ajpc.2024.100918)
Supplement: Supplementary file 1 [file mmc1.docx]

**Supplemental Materials**

**Supplemental Methods**

**Assessment of vascular ageing manifestations**

Carotid pulse pressure was calculated by calibrating the carotid distension waveform obtained from echotracking using brachial mean diastolic blood pressure. Brachial blood pressure was measured continuously during vascular echotracking with an oscillometric device (Omron 705 C, Kyoto, Japan) and the immediate blood pressure reading after vascular evaluation was used for carotid pulse pressure calculation.

The carotid distensibility coefficient (DC) was calculated according to the following equation: ΔLCSA / LCSA ∙ PP, where LCSA is diastolic lumen cross-sectional area, ΔLCSA is change in lumen area, and PP is carotid pulse pressure. YEM was calculated as 3 ∙ (1+LCSA / WCSA) / DC, where WCSA is the wall cross-sectional area. WCSA was calculated as π(De^2^-Di^2^)/4(mm^2^), where De is external diameter and Di internal diameter. ^1^ Local carotid pulse wave velocity, which has shown a good correlation with the gold-standard carotid-femoral pulse wave velocity, ^2^ was estimated from the Bramwell and Hill equation. ^3^ Beta stiffness index was calculated as Ln(Ps/Pd) / (Ds-Dd)/Dd, where Ps is local systolic pressure, Pd local diastolic pressure, Ds systolic diameter and Dd diastolic diameter. From IMT and carotid diameter, lumen diameter (LD) was calculated using the following formula: LD=carotid diameter-(2∙IMT) in mm. Circumferential wall stress was then calculated according to Laplace’s law as P*(r/w), where P is transmural mean arterial pressure, r is lumen radius and w is wall thickness. Mean blood pressure was used as representative of transmural mean arterial pressure.^4^ The reproducibility of carotid echotracking measurements has been reported previously.^5^

A carotid plaque was defined as a focal thickening encroaching into the carotid lumen of more than 1.5 mm (as measured from the intima‐lumen interface to the media‐adventitia interface) or at least 50% of the surrounding IMT. Carotid IMT was measured along a plaque‐free segment of the common carotid artery during end‐diastole. IMT was defined as the distance between the lumen‐intima and media adventitia interfaces of the far (posterior) wall.

**Optimal number of clusters of vascular ageing**

Number of clusters:

* Among all indices:

*12 proposed 3 as the best number of clusters (46%)

* 7 proposed 2 as the best number of clusters (27%)

* 4 proposed 5 as the best number of clusters (15%)

* 2 proposed 6 as the best number of clusters (8%)

* 1 proposed 7 as the best number of clusters (4%)

***** Conclusion *****

* According to the majority rule, the optimal number of clusters is 3.

**Propensity score matching**

We determined each participant’s propensity to receive antihypertensive medication during follow-up using multivariate logistic regression adjusted for systolic and diastolic blood pressure, age, sex, BMI, smoking, alcohol intake, education, family history of hypertension, kidney function, previous CVD, type 2 diabetes at baseline. Those treated and those untreated were then matched (ratio 1:4) based on their respective propensity scores using optimal matching, which minimizes global propensity score difference between matched pairs by considering the over-all set of matches. Propensity scores and covariates were considered balanced when standardized mean difference (SMD) <0.25 ^7^ and a variance ratio close to 1.0.^8^ Then, the association between clusters of vascular ageing manifestations and incident hypertension was estimated via conditional logistic regression to account for the matching design.

**Supplemental Table 1**. Baseline characteristics between included and excluded participants for the cross-sectional and prospective analyses.

|  | **Cross-sectional analysis** | | **Prospective analysis** | | |
| --- | --- | --- | --- | --- | --- |
|  | **Included,**  **N = 9,096** | **Excluded,**  **N = 1,061** | **Included**  **N=5,310** | **Excluded, N=510** | |
|  |  |  |  | **Lost-to-follow-up**  **N=283** | **Missing BP medication, N=227** |
| Age, years | 60 (6) | 60 (6) | 59 (6) | 57.4 (5.4) | 59.3 (6) |
| Sex, male | 5,556 (61%) | 634 (60%) | 3,209 (60%) | 169 (60%) | 143 (63%) |
| BMI, kg/m^2^ | 25.1 (3.6) | 25.9 (4.4) | 24.5 (3.4) | 25.0 (3.8) | 24.7 (3.5) |
| HDL, mg/dL | 58 (15) | 57 (15) | 59 (15) | 56 (16) | 57 (15) |
| Total cholesterol, mg/dL | 221 (36) | 224 (38) | 222 (35) | 217 (37) | 223 (38) |
| Systolic BP, mmHg | 131 (16) | 133 (17) | 123 (10) | 124 (10) | 123 (10) |
| Diastolic BP, mmHg | 76 (10) | 77 (10) | 72 (7) | 73 (8) | 72 (7) |
| History of CVD | 195 (2.1%) | 35 (3.6%) | 1 (<0.01%) | 4 (1.4) | 61 (27%) |
| Type 2 diabetes | 369 (4.1%) | 68 (6.7%) | 117 (2.2%) | 11 (3.9%) | 6 (2.6%) |
| Hypertension | 3,276 (36%) | 424 (43%) | 0 (0%) | - | - |
| Alcohol intake |  |  |  |  |  |
| Abstainer | 1,108 (12%) | 133 (13%) | 630 (12%) | 61 (22%) | 33 (15%) |
| Occasional intake | 5,738 (63%) | 599 (59%) | 3,531 (66%) | 172 (61%) | 150 (66%) |
| Regular intake | 2,250 (25%) | 288 (28%) | 1,221 (23%) | 50 (18%) | 44 (19%) |
| Smoking |  |  |  |  |  |
| Non-smoker | 4,727 (52%) | 468 (45%) | 2,855 (53%) | 144 (51%) | 115 (51%) |
| Former | 2,936 (32%) | 372 (36%) | 1,682 (31%) | 80 (28%) | 67 (30%) |
| Active | 1,433 (16%) | 201 (19%) | 859 (16%) | 59 (21%) | 45 (20%) |

Values are mean (SD) and n (%). BMI, body mass index; BP, blood pressure; CVD, cardiovascular disease; HDL, high-density lipoprotein cholesterol.

**Supplemental Table 2.** Levels and distribution of individual vascular ageing manifestations by clusters of vascular ageing manifestation at baseline.

| **Vascular ageing manifestations** | **Overall,**  **N = 9,096** | **HVA,**  **N = 4,326** | **ART,**  **N = 2,274** | **ATH,**  **N = 2,496** |
| --- | --- | --- | --- | --- |
| cPWV, m/s | 7.35 (1.46) | 6.46 (0.76) | 9.14 (1.30) | 7.25 (0.96) |
| Young’s elastic modulus, kPa | 498 (227) | 389 (104) | 785 (245) | 426 (117) |
| Distensibility coefficient, kPa-1*10-3 | 22 (8) | 27 (7) | 13 (3) | 21 (6) |
| Β stiffness index | 9.7 (3.6) | 7.9 (1.8) | 13.9 (4.0) | 9.1 (2.3) |
| Central pulse pressure, mmHg | 46 (11) | 41 (8) | 52 (12) | 49 (10) |
| CWS, kPa | 59 (14) | 60 (13) | 66 (16) | 52 (11) |
| WCSA, μm²*106 | 13.2 (3.1) | 11.4 (1.7) | 12.9 (2.5) | 16.7 (2.7) |
| Intima media thickness, micrometers | 640 (116) | 582 (78) | 613 (91) | 767 (92) |
| Plaque presence | 1,064 (12%) | 232 (5.4%) | 281 (12%) | 551 (22%) |
| Diameter, millimeters | 7.17 (0.71) | 6.81 (0.53) | 7.27 (0.67) | 7.69 (0.67) |

Values are mean (SD) and n (%).; ART, arteriosclerosis; ATH, atherosclerosis; cPWV, carotid pulse wave velocity; CWS, cross-sectional wall stress; HVA, healthy vascular ageing; WCSA, wall cross-sectional area.

**Supplemental Table 3.** Clinical characteristics of study participants by clusters of vascular ageing manifestations.

|  | **HVA,**  **N = 4,326** | **ART,**  **N = 2,274** | **ATH,**  **N = 2,496** |
| --- | --- | --- | --- |
| Age, years | 56 (53, 61) | 60 (56, 65) | 62 (57, 67) |
| Sex, male | 2,449 (57%) | 1,398 (61%) | 1,709 (68%) |
| BMI, kg/m^2^ | 24.2 (3.4) | 25.8 (3.7) | 25.9 (3.6) |
| High education | 1,781 (42%) | 771 (34%) | 994 (40%) |
| Type 2 diabetes | 94 (2.2%) | 130 (5.7%) | 145 (5.8%) |
| History of CVD | 61 (1.4%) | 61 (2.7%) | 73 (2.9%) |
| HDL, mg/dL | 60 (15) | 57 (15) | 57 (15) |
| Total cholesterol, mg/dL | 220 (35) | 223 (37) | 220 (37) |
| Smoking |  |  |  |
| Non-smoking | 2,291 (53%) | 1,226 (54%) | 1,210 (48%) |
| Former | 1,286 (30%) | 737 (32%) | 913 (37%) |
| Regular | 749 (17%) | 311 (14%) | 373 (15%) |
| Alcohol intake |  |  |  |
| Non-alcohol drinker | 533 (12%) | 294 (13%) | 281 (11%) |
| Occasional | 2,881 (67%) | 1,428 (63%) | 1,429 (57%) |
| Regular | 912 (21%) | 552 (24%) | 786 (31%) |
| Lipid lowering drugs | 439 (10%) | 366 (16%) | 431 (17%) |
| eGFR, mL/min/1.73 m^2^ | 86 (12) | 82 (13) | 82 (12) |
| Heart rate, bpm | 61 (9) | 64 (9) | 61 (9) |

Values are mean (SD) and n (%). ART, arteriosclerosis; ATH, atherosclerosis; BMI, body mass index; CVD, cardiovascular disease; eGFR, estimated glomerular filtration rate. HDL, high-density cholesterol; HVA, healthy vascular ageing.

**Supplemental Table 4.** Association between clusters of vascular ageing manifestations, the covariates and systolic and diastolic blood pressure: cross-sectional analysis

|  | **Systolic BP, mm Hg** | |  | **Diastolic BP, mm Hg** | |  |
| --- | --- | --- | --- | --- | --- | --- |
|  | **β** | **95% CI** | **p** | **β** | **95% CI** | **p** |
| HVA | Ref. |  |  | Ref. |  |  |
| ART | +13.35 | 12.58 – 14.13 | <0.001 | +2.83 | 2.35 – 3.32 | <0.001 |
| ATH | +8.64 | 7.87 – 9.41 | <0.001 | +1.58 | 1.10 – 2.07 | <0.001 |
| Age, years | 0.2 | 0.15 – 0.26 | <0.001 | -0.09 | -0.12 – -0.05 | <0.001 |
| Sex, male | 4.19 | 3.46 – 4.92 | <0.001 | 2.98 | 2.53 – 3.44 | <0.001 |
| BMI, kg/m^2^ | 0.59 | 0.50 – 0.68 | <0.001 | 0.36 | 0.31 – 0.42 | <0.001 |
| High education |  |  |  |  |  |  |
| No | Ref. |  | Ref. |  |  |  |
| Yes | -1.37 | -1.99 – -0.75 | <0.001 | -0.41 | -0.80 – -0.02 | 0.040 |
| Type 2 diabetes | 0.86 | -0.67 – 2.40 | 0.270 | -0.21 | -1.18 – 0.75 | 0.666 |
| HDL, mg/dL | 0.06 | 0.04 – 0.09 | <0.001 | 0.02 | -0.01 – 0.04 | 0.003 |
| Total cholesterol, mg/dL | 0.01 | 0.00 – 0.02 | 0.009 | 0.01 | 0.01 – 0.02 | <0.001 |
| Non-smoking | Ref. |  |  |  |  |  |
| Former | 0.07 | -0.60 – 0.75 | 0.836 | 0.04 | -0.38 – 0.47 | 0.846 |
| Regular | -0.25 | -1.13 – 0.63 | 0.575 | -0.28 | -0.83 – 0.27 | 0.322 |
| Non-alcohol drinker | Ref. |  |  |  |  |  |
| Occasional | -0.42 | -1.35 – 0.52 | 0.382 | 0.06 | -0.53 – 0.65 | 0.847 |
| Regular | 1.60 | 0.52 – 2.69 | 0.004 | 1.00 | 0.32 – 1.68 | 0.004 |
| Lipid lowering drugs | 0.78 | 0.04– 1.70 | 0.092 | 0.31 | -0.26 – 0.89 | 0.289 |
| eGFR, mL/min/1.73 m^2^ | -0.01 | -0.04 – 0.01 | 0.369 | -0.02 | -0.04 – 0.02 | 0.028 |
| Heart rate, bpm | 0.19 | 0.15 – 0.22 | <0.001 | 0.21 | 0.18 – 0.23 | <0.001 |

Coefficients for were estimated with multivariate linear regression analysis adjusted for the variables listed in the Table. ART, arteriosclerosis; ATH, atherosclerosis; BP, blood pressure; BMI, body mass index; eGFR, estimated glomerular filtration rate; HDL, high density lipoprotein cholesterol; HVA, healthy vascular ageing.

**Supplemental Table 5.** Association between clusters of vascular ageing manifestations and incident hypertension stratified by age.

|  | **1^st^ age tertile**  **N=2,063** | **2^nd^ age tertile**  **N=1,804** | **3^rd^ age tertile**  **N=1,443** |
| --- | --- | --- | --- |
|  | **Range=50-55** | **Range=55-61** | **Range=61-75** |
|  | OR (95% CI) | | |
| HVA | Ref. | Ref. | Ref. |
| ART | 1.63 (1.13;2.33) | 1.37 (0.96;1.95) | 1.08 (0.73;1.58) |
| ATH | 1.85 (1.37;2.86) | 1.83 (1.32;2.55) | 1.55 (1.13;2.12) |
|  | *P value for age interaction=0.018* | | |

OR and 95% CI were estimated with multivariate logistic regression and adjusted for age, body mass index, diabetes, smoking, education, alcohol intake, total cholesterol, high-density cholesterol, lipid lowering drugs, estimated glomerular filtration rate, and heart rate. ART, arteriosclerosis; ATH, atherosclerosis; HVA, healthy vascular ageing

**Supplemental Table 6.** Association between clusters of vascular ageing manifestations and incident hypertension stratified by sex.

|  | **Men**  **N=3,168** | **Women**  **N=2,142** |
| --- | --- | --- |
|  | OR (95% CI) | OR (95% CI) |
| HVA | Ref. | Ref. |
| ART | 1.10  (0.84 ;1.44) | 1.82  (1.28;2.55) |
| ATH | 1.70  (1.34;2.16) | 1.61  (1.14;2.26) |
|  | *P value for sex interaction= 0.023* | |

OR and 95% CI were estimated with multivariate logistic regression analysis and adjusted for age, body mass index, diabetes, smoking, education, alcohol intake, total cholesterol, high-density cholesterol, lipid lowering drugs, estimated glomerular filtration rate, and heart rate. ART, arteriosclerosis; ATH, atherosclerosis; CI, confidence interval; HVA, healthy vascular ageing.

**Supplemental Table 7.** Secondary analysis on the association between individual vascular ageing manifestations with blood pressure at baseline, prevalent and incident hypertension.

|  | **Cross-sectional analysis** | | | **Prospective analysis** |
| --- | --- | --- | --- | --- |
| **Individual**  **manifestations** | **Systolic BP,**  **mm Hg*** | **Diastolic BP,**  **mm Hg*** | **Prevalent hypertension†** | **Incident hypertension†** |
|  | **β (95% CI)** | | **OR (95% CI)** | |
| cPWV, m/s, per  1 SD increment | 4.47  (4.26;4.68) | 1.21  (1.08;1.35) | 2.02  (1.91;2.13) | 1.09  (1.00;1.17) |
| Young’s elastic  modulus, kPa,  per 1 SD increment | 0.025  (0.023:0.026) | 0.007  (0.006;0.008) | 1.83  (1.73;1.93) | 1.01  (0.94;1.09) |
| DC, kPa-1*10-3, per  1 SD increment | -0.79  (-0.83;-0.75) | -0.19  (-0.22;-0.17) | 0.48  (0.46;0.51) | 0.89  (0.81;0.97) |
| β stiffness index,  per 1 SD increment | 0.56  (0.47;0.65) | -0.25  (-0.30;-0.20) | 1.29  (1.23;1.35) | 1.20  (1.02;1.43) |
| cPP, mmHg,  per 1 SD increment | 1.13  (1.11;1.15) | 0.11  (0.09;0.13) | 4.12  (3.84;4.43) | 1.21  (1.12;1.31) |
| CWS, kPa, per 1 SD  increment | 0.49  (0.47;0.51) | 0.31  (0.30;0.32) | 1.84  (1.74;1.94) | 1.04  (0.95;1.13) |
| WCSA, μm²*106,  per 1 SD increment | 1.06  (0.95;1.18) | 0.42  (0.35;0.48) | 1.66  (1.58;1.75) | 1.37  (1.28;1.49) |
| IMT, µm, per 1 SD  increment | 0.018  (0.015;0.021) | 0.006  (0.005;0.008) | 1.43  (1.36;1.50) | 1.38  (1.28;1.49) |
| Plaque presence | 3.52  (2.52;4.53) | 0.80  (0.20;1.40) | 1.90  (1.66;2.19) | 1.34  (1.03;1.72) |
| Diameter, mm, per  1 SD increment | 5.36  (4.85;5.86) | 2.21  (1.91;2.53) | 1.77  (1.67;1.86) | 1.33  (1.22;1.45) |
| **Clusters** |  |  |  |  |
| HVA | Ref. | Ref. | Ref. | Ref. |
| ART | +13.35 (12.58 – 14.13) | +2.83  (2.35 – 3.32) | 3.94  (3.50;4.45) | 1.34  (1.08;1.65) |
| ATH | +8.64  (7.87 – 9.41) | +1.58  (1.10 – 2.07) | 2.69  (2.38;3.04) | 1.70  (1.40;2.07) |

^*^Coefficients estimated with multivariate linear regression. ^†^OR and 95% CI estimated with multivariate logistic regression. All analyses were adjusted for age, body mass index, diabetes, smoking, education, alcohol intake, total cholesterol, high-density cholesterol, lipid lowering drugs, estimated glomerular filtration rate and heart rate. ART, arteriosclerosis; ATH, atherosclerosis; CI, confidence interval; cPP; central pulse pressure; cPWV, carotid pulse wave velocity; CWS, cross-sectional wall stress; DC, distensibility coefficient; HVA, healthy vascular ageing; IMT, intima media thickness; WCSA, wall cross-sectional area.

**Supplemental Table 8.** Performances of the propensity score of antihypertensive medication at follow-up after optimal matching.

|  | **Means Treated**  **N=3,004** | **Means Control**  **N=751** | **SMD** | **VR** |
| --- | --- | --- | --- | --- |
| Propensity score | 0.1797 | 0.166 | 0.168 | 1.289 |
| *Covariates* |  |  |  |  |
| SBP, mmHg | 126.4 | 125.9 | 0.061 | 1.190 |
| DBP, mmHg | 74.5 | 74.1 | 0.061 | 1.254 |
| Age, years | 59.8 | 59.3 | 0.084 | 1.064 |
| Male, % | 0.649 | 0.639 | 0.020 | . |
| Family history of hypertension, % | 0.371 | 0.355 | 0.032 | . |
| Non-smoking, % | 0.48 | 0.50 | -0.041 | . |
| Former smoker, % | 0.33 | 0.33 | 0.010 | . |
| Active smoker, % | 0.19 | 0.17 | 0.040 | . |
| Alcohol abstain, % | 0.11 | 0.12 | -0.015 | . |
| Occasional alcohol intake, % | 0.64 | 0.64 | 0.000 | . |
| Regular intake, % | 0.25 | 0.25 | 0.010 | . |
| eGFR, mL/min/1.73 m^2^ | 83.39 | 83.35 | -0.076 | 1.135 |
| BMI, kg/m^2^ | 25.3 | 25.0 | 0.073 | 1.204 |

Data are presented as mean or frequency. Optimal matching of propensity scores was performed with a 1:4 treated-to-control ratio. BMI, body mass index; DBP, diastolic blood pressure; eGFR, estimated glomerular filtration rate; SBP, systolic blood pressure; SMD, standard mean difference; VR, variance ratio.

**Supplemental Table 9.** Association between clusters of vascular ageing manifestations and incident hypertension after optimal matching of propensity scores.

|  | **Optimal matching analysis**  **N=3,755** | **Main analysis**  **N=5,310** |
| --- | --- | --- |
|  | OR (95% CI) | |
| HVA | Ref. | Ref. |
| ART | 1.24 (1.00;1.54) | 1.34 (1.08;1.65) |
| ATH | 1.68 (1.39;2.03) | 1.70 (1.40;2.07) |

Conditional logistic regression was used for the optimal matching analysis, and binary logistic regression for the main analysis, respectively. ART, arteriosclerosis; ATH, atherosclerosis; HVA, healthy vascular ageing.

**Supplemental Table 10.** Sensitivity analysis of the association between clusters of vascular ageing manifestations and prevalent hypertension.

|  | **Free of previous CVD**  **n/N=3,147/8,901*** | **Free of traditional risk factors ^†^**  **n/N=1,242/4,339** |
| --- | --- | --- |
|  | OR 95% CI | |
| HVA | Ref. | Ref. |
| ART | 4.13  (3.66;4.66) | 4.41  (3.71;5.24) |
| ATH | 2.68  (2.37;3.02) | 2.99  (2.49;3.59) |

n/N refers to prevalent hypertension cases relative to the number of participants in the studied sample. OR and 95% CI were estimated with multivariate logistic regression. ^*^Adjusted for age, sex, BMI, education, diabetes, smoking, alcohol intake, lipid-lowering drugs, cholesterol levels, eGFR, and heart rate. ^†^The subsample excluded those with BMI >30, active smokers, regular alcohol drinkers, n type 2 diabetes, use of lipid-lowering drugs and eGFR <60 mL/min/1.73 m^2^).Adjusted for age, sex, education, and heart rate. ART, arterial stiffness; ATH, atherosclerosis; BMI, body mass index; eGFR, estimated glomerular filtration rate; HVA, healthy vascular ageing.

**Supplemental Table 11.** Sensitivity analysis of the association between clusters of vascular ageing manifestations and incident hypertension.

|  | **Blood pressure categories at baseline***§ | | | |
| --- | --- | --- | --- | --- |
|  | **Optimal**  <120 and <80 mmHg  **n/N=146/1,787** | **Normal**  120-129 and 80-84 mmHg  **n/N=275/1,945** | | **High normal**  130-139 and/or 85-89 mmHg  **n/N=333/1,578** |
|  | **OR (95% CI)** | | | |
| HVA | Ref. | Ref. | | Ref. |
| ART | 1.37  (0.76;2.35) | 0.94  (0.66:1.34) | | 1.21  (0.89;1.66) |
| ATH | 2.03  (1.34;3.07) | 1.31  (0.95;1.81) | | 1.54  (1.12;2.10) |
|  | P for interaction = 0.083 | | | |
|  | **Free of previous CVD and CVD event during follow-up** | | | |
|  | **Incident hypertension ***§  **n/N=610/5,065** | | **Per number of antihypertensive medications at follow-up (0, 1, 2 and more) ^†^**§ | |
|  | **OR 95% CI** | | | |
| HVA | Ref. | | Ref. | |
| ART | 1.58 (1.26-1.98) | | 1.51 (1.20-1.90) | |
| ATH | 2.01 (1.64-2.47) | | 1.86 (1.50-2.31) | |
|  | **Free of traditional risk factors**‡ | | | |
|  | **Incident hypertension ***\|\| **n/N=336/1,838** | | **Per number of antihypertensive medications at follow-up (0, 1, 2 and more)†**\|\| | |
|  | **OR 95% CI** | | | |
| HVA | Ref. | | Ref. | |
| ART | 1.41 (1.03;1.91) | | 1.34 (0.98;1.84) | |
| ATH | 1.61 (1.20;2.15) | | 1.51 (1.12;2.04) | |

BP categories followed the 2023 ESH guidelines.^9^ n/N refers to incident hypertension cases relative to the number of participants in the considered category. **^*^**Binary logistic regression. **^†^**Ordinal logistic regression. ‡The subsample excluded those with BMI >30, active smokers, regular alcohol drinkers, type 2 diabetes, use of lipid-lowering drugs and eGFR <60 mL/min/1.73 m^2^). § Adjusted for age, sex, BMI, education, diabetes, smoking, alcohol intake, lipid-lowering drugs, cholesterol levels, eGFR, and heart rate. ||Adjusted for age, sex, education, and heart rate. ART, arteriosclerosis; ATH, atherosclerosis; BMI, body mass index; CVD, cardiovascular disease; eGFR, estimated glomerular filtration rate; HVA, healthy vascular ageing.

**Supplemental Table 12.** Sensitivity analysis of association between clusters of vascular ageing manifestations with prevalent and incident hypertension after adjusting for brachial pulse pressure.

|  | **Prevalent hypertension*** | | **Incident hypertension** | |
| --- | --- | --- | --- | --- |
|  | **Main analysis** | **Adjusted for pPP** | **Main analysis** | **Adjusted for pPP** |
|  | **OR (95%CI)** | **OR (95%CI)** | **OR (95%CI)** | **OR (95%CI)** |
| HVA | Ref. | Ref. | Ref. | Ref. |
| ART | 3.94  (3.50;4.45) | 1.55  (1.35;1.79) | 1.34  (1.08;1.65) | 1.25  (1.01;1.55) |
| ATH | 2.69  (2.38;3.04) | 1.39  (1.21;1.60) | 1.70  (1.40;2.07) | 1.63  (1.33;1.98) |

OR and 95% CI were estimated with multivariate logistic regression. Adjusted for age, sex, body mass index, diabetes, smoking, education, alcohol intake, lipid-lowering drugs, total cholesterol, high-density cholesterol, estimated glomerular filtration rate, and heart rate. ^*^Additional adjustment for history of cardiovascular disease. ART, arteriosclerosis; ATH, atherosclerosis, HVA, healthy vascular ageing; pPP; peripheral pulse pressure.

**Supplemental Table 13**. Clustering validity indices and optimal number of clusters in each test.

|  |  | Number of clusters | | | | | |  |
| --- | --- | --- | --- | --- | --- | --- | --- | --- |
|  | Test | 2 | 3 | 4 | 5 | 6 | 7 | Optimal clusters |
| 1 | KL | 1.585 | 3.365 | 0.935 | 0.912 | 2.157 | 1.234 | 3 |
| 2 | CH | 5,286.964 | 5,914.321 | 5,207.098 | 5,115.927 | 5,480.434 | 5,369.001 | 3 |
| 3 | Hartigan | 3,998.360 | 1,565.332 | 1,681.695 | 2,003.787 | 1,119.864 | 980.227 | 3 |
| 4 | CCC | 163.507 | 158.343 | 15.799 | -86.419 | -172.324 | -162.872 | 2 |
| 5 | Scott | 76,830.51 | 84,896.32 | 88,727.87 | 93,718.90 | 100,129.20 | 102,140.40 | 3 |
| 6 | Marriot | 1.05813E+49 | 9.01857E+48 | 1.01099E+49 | 8.66358E+48 | 5.76775E+48 | 6.16E+62 | 6 |
| 7 | TrCovW | 6.08554E+15 | 2.41072E+15 | 1.7865E+15 | 1.31291E+15 | 8.48884E+14 | 6.20119E+14 | 3 |
| 8 | TraceW | 329281446 | 222288581 | 187039505 | 155543262 | 125306355 | 110414277 | 3 |
| 9 | Friedman | 10,710.050 | 10,969.360 | 11,260.660 | 11,706.840 | 12,229.550 | 12,278.750 | 6 |
| 10 | Rubin | 18.369 | 27.210 | 32.338 | 38.886 | 48.270 | 54.780 | 3 |
| 11 | Cindex | 0.13 | 0.101 | 0.089 | 0.078 | 0.138 | 0.129 | 5 |
| 12 | DB | 0.818 | 1.129 | 1.245 | 1.181 | 1.049 | 1.001 | 2 |
| 13 | Silhouette | 0.581 | 0.328 | 0.268 | 0.272 | 0.278 | 0.265 | 2 |
| 14 | Duda | 0.606 | 0.638 | 0.533 | 0.474 | 0.602 | 0.53 | 3 |
| 15 | Pseudot2 | 4,978.334 | 2,352.681 | 3,084.711 | 705.076 | 1,169.205 | 2,110.815 | 5 |
| 16 | Beale | 4.364 | 3.810 | 5.897 | 7.467 | 4.452 | 5.949 | 3 |
| 17 | Ratkowsky | 0.218 | 0.256 | 0.265 | 0.274 | 0.254 | 0.241 | 5 |
| 18 | Ball | 164640723 | 74096194 | 46759876 | 31108652 | 20884393 | 15773468 | 3 |
| 19 | Ptbiserial | 0.631 | 0.435 | 0.402 | 0.374 | 0.379 | 0.377 | 2 |
| 20 | Frey | 4.883 | 1.104 | 0.708 | -0.225 | 0.312 | 0.873 | 3 |
| 21 | McClain | 0.062 | 0.643 | 1.026 | 1.468 | 1.448 | 1.544 | 2 |
| 22 | Dunn | 0.008 | 0.003 | 0.003 | 0.003 | 0.006 | 0.006 | 2 |
| 23 | Hubert | Graphical method | | | | | | 3 |
| 24 | SDindex | 0.022 | 0.023 | 0.024 | 0.023 | 0.027 | 0.027 | 2 |
| 25 | Dindex | Graphical method | | | | | | 5 |
| 26 | SDbw | 1.289 | 1.192 | 0.981 | 0.937 | 0.809 | 0.76 | 7 |


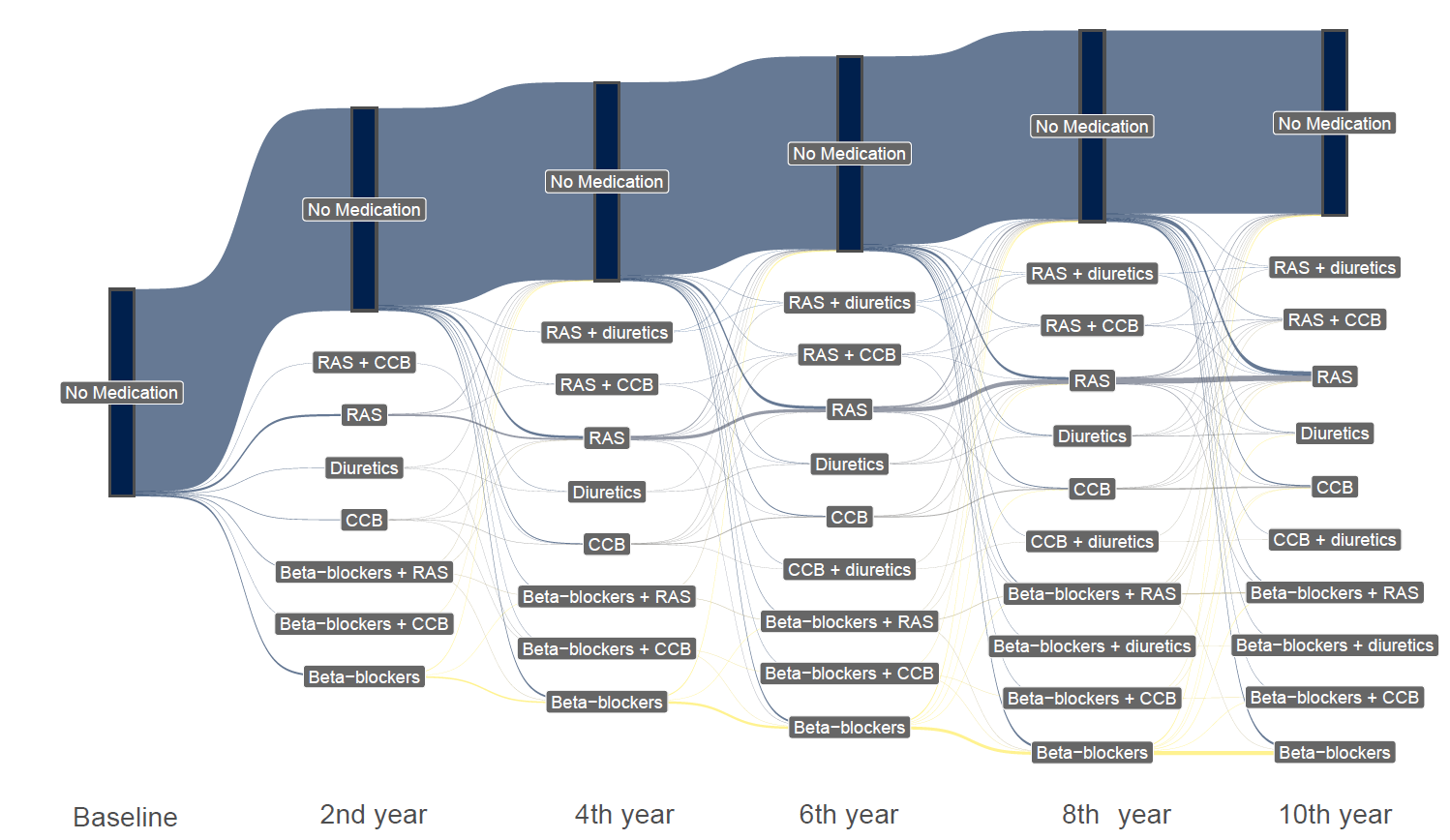


**Supplemental Figure 1.** Sankey diagram showing the distribution and changes of class of antihypertensive medications during follow-up. CCB, calcium channel blockers; RAS, renin-angiotensin system.


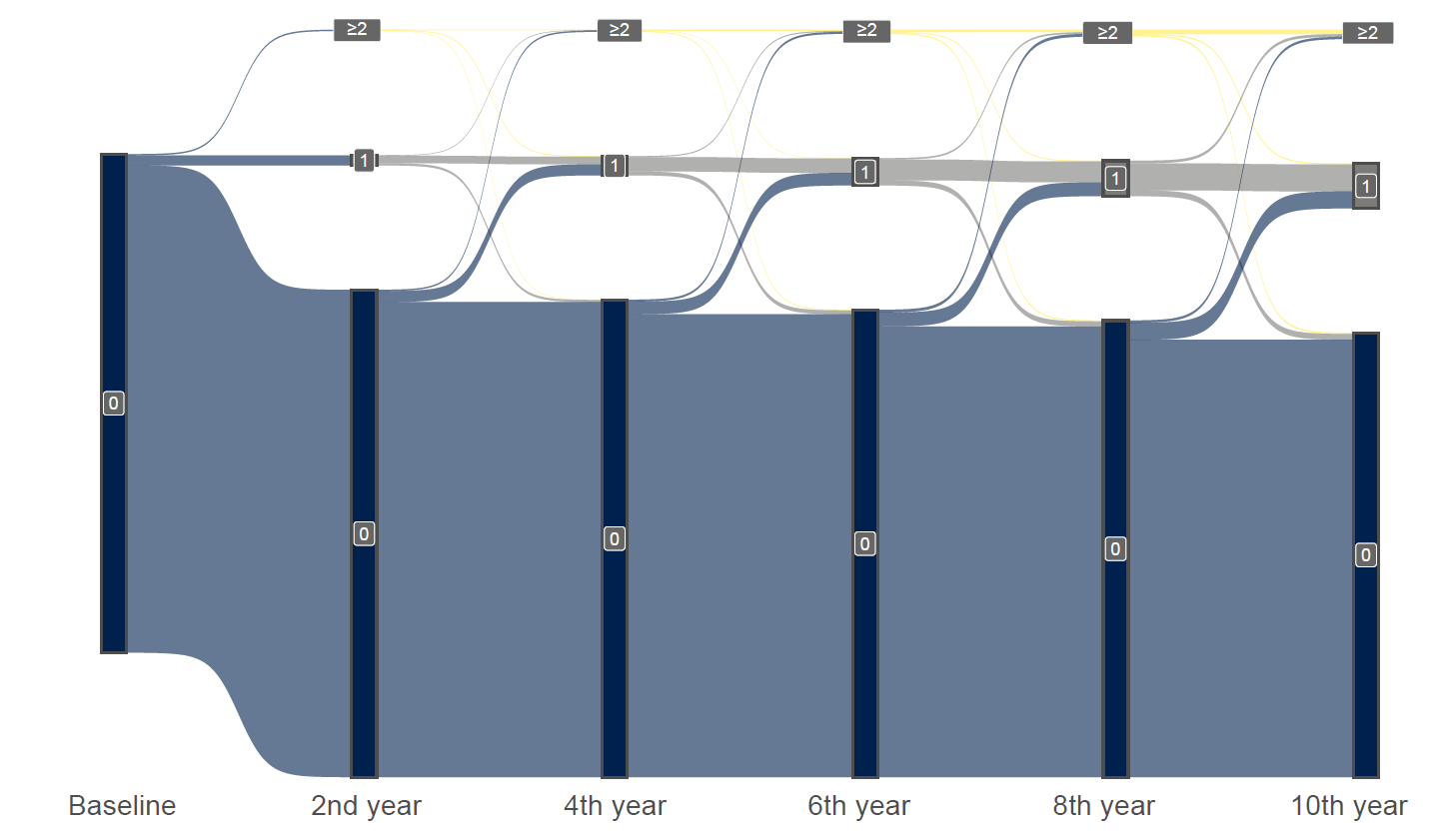


**Supplemental Figure 2.** Sankey diagram showing the distribution of the number of antihypertensive medications (0,1, ≥2) during follow-up.


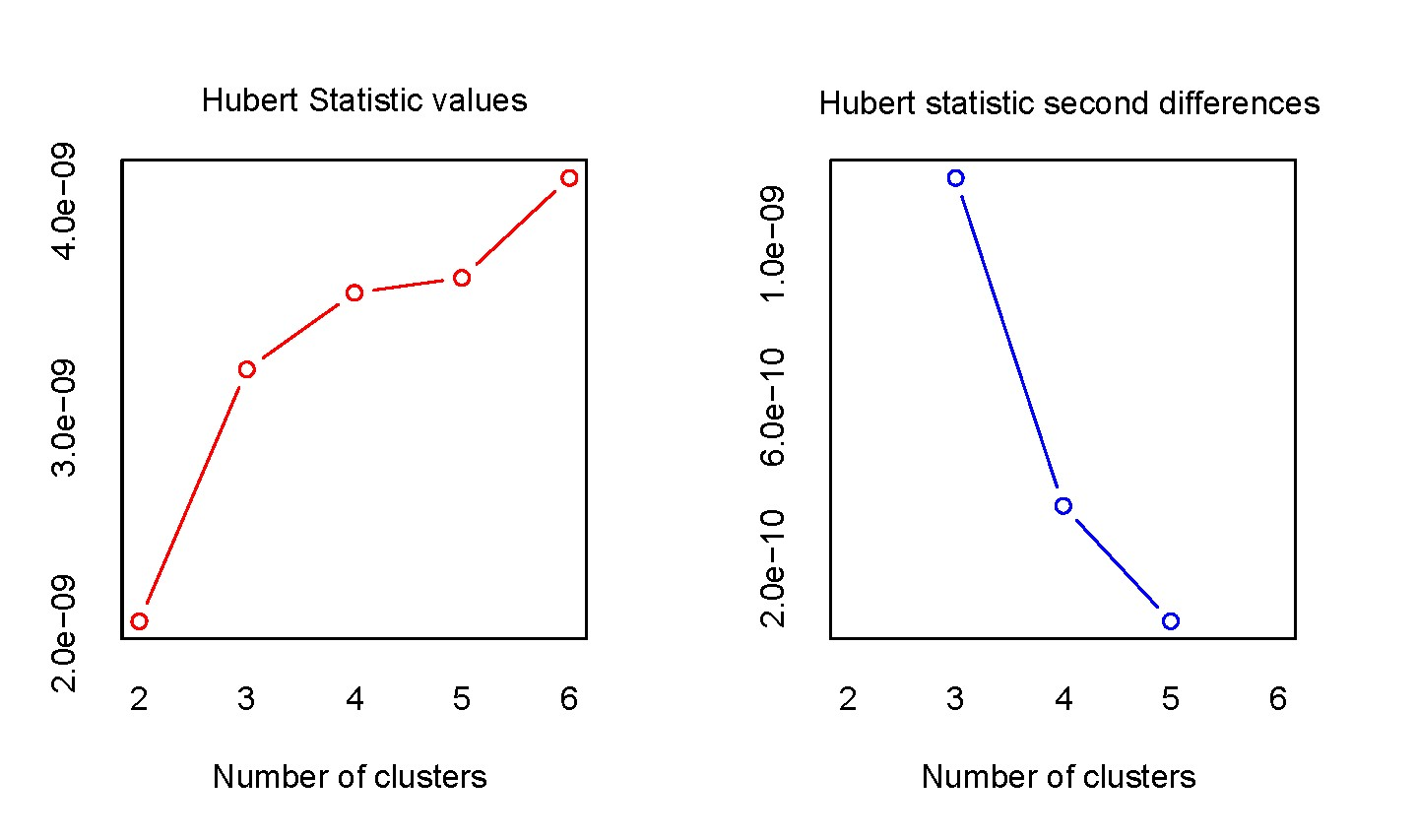

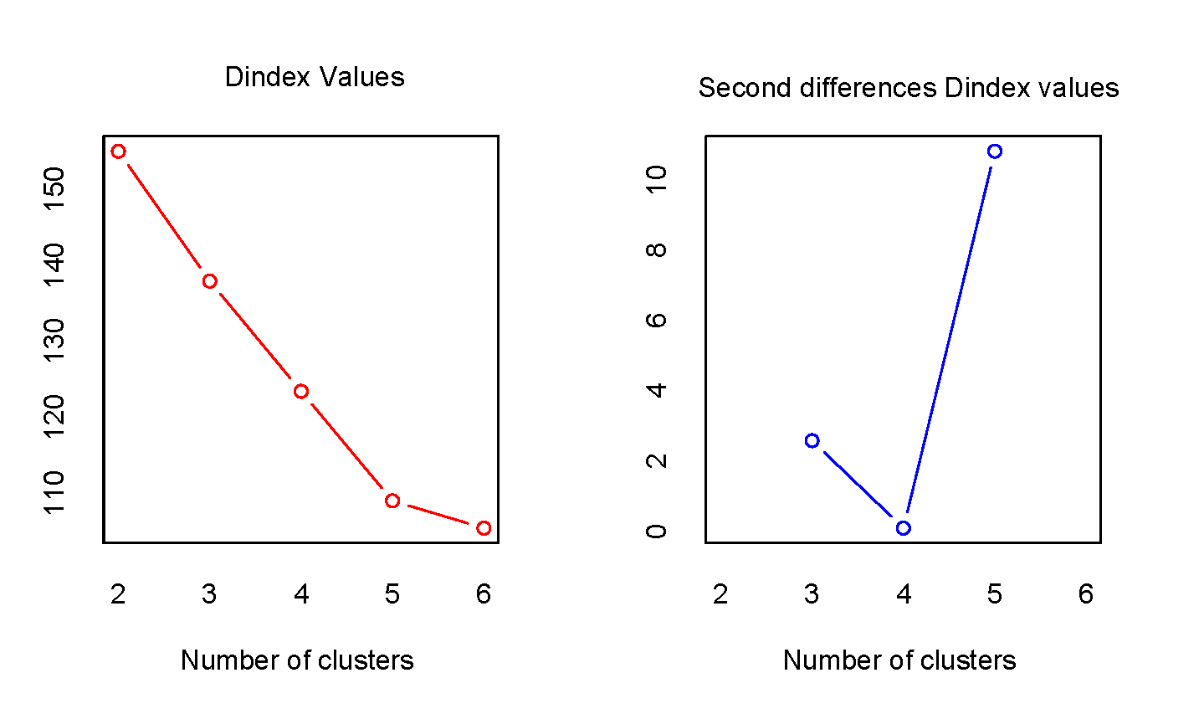


**Supplemental Figure 3.** Graphical methods for determining the best number of clusters.

**References**

1. Laurent S, Cockcroft J, Van Bortel L, Boutouyrie P, Giannattasio C, Hayoz D, Pannier B, Vlachopoulos C, Wilkinson I, Struijker-Boudier H, et al. Expert consensus document on arterial stiffness: methodological issues and clinical applications. *Eur Heart J*. 2006;27:2588–605.

2. Vriz O, Driussi C, La Carrubba S, Di Bello V, Zito C, Carerj S, Antonini-Canterin F. Comparison of sequentially measured Aloka echo-tracking one-point pulse wave velocity with SphygmoCor carotid-femoral pulse wave velocity. *SAGE Open Med*. 2013;1:2050312113507563.

3. Bramwell JC, Hill A V. Velocity of transmission of the pulse-wave: and elasticity of arteries. *The Lancet*. 1922;199:891–892.

4. Geijselaers SL, Sep SJ, Schram MT, van Boxtel MP, van Sloten TT, Op Het Roodt J, Henry RM, Reesink KD, Schaper NC, Dagnelie PC, et al. Carotid circumferential wall stress is not associated with cognitive performance among individuals in late middle age: The Maastricht Study. *Atherosclerosis*. 2018;276:15–22.

5. Bianchini E, Bozec E, Gemignani V, Faita F, Giannarelli C, Ghiadoni L, Demi M, Boutouyrie P, Laurent S. Assessment of carotid stiffness and intima-media thickness from ultrasound data: comparison between two methods. *J Ultrasound Med*. 2010;29:1169–75.

6. Sharma A, Zheng Y, Ezekowitz JA, Westerhout CM, Udell JA, Goodman SG, Armstrong PW, Buse JB, Green JB, Josse RG, et al. Cluster Analysis of Cardiovascular Phenotypes in Patients With Type 2 Diabetes and Established Atherosclerotic Cardiovascular Disease: A Potential Approach to Precision Medicine. *Diabetes Care*. 2022;45:204–212.

7. Stuart EA, Rubin DB. Best practices in quasi-experimental designs. *Best practices in quantitative methods*. 2008;155–176.

8. Rubin DB. Using propensity scores to help design observational studies: application to the tobacco litigation. *Health Serv Outcomes Res Methodol*. 2001;2:169–188.

9. Mancia G, Kreutz R, Brunström M, Burnier M, Grassi G, Januszewicz A, Muiesan ML, Tsioufis K, Agabiti-Rosei E, Algharably EAE, et al. 2023 ESH Guidelines for the management of arterial hypertension The Task Force for the management of arterial hypertension of the European Society of Hypertension: Endorsed by the International Society of Hypertension (ISH) and the European Renal Association (ERA). *J Hypertens*. 2023;41:1874–2071.
